# Restoration of Physiologically Responsive Low-Density Lipoprotein Receptor-Mediated Endocytosis in Genetically Deficient Induced Pluripotent Stem Cells

**DOI:** 10.1038/srep13231

**Published:** 2015-08-26

**Authors:** Venkat M. Ramakrishnan, Jeong-Yeh Yang, Kevin T. Tien, Thomas R. McKinley, Braden R. Bocard, John G. Maijub, Patrick O. Burchell, Stuart K. Williams, Marvin E. Morris, James B. Hoying, Richard Wade-Martins, Franklin D. West, Nolan L. Boyd

**Affiliations:** 1 Cardiovascular Innovation Institute, University of Louisville School of Medicine and Jewish Hospital, Louisville, Kentucky 40202, USA; 2 Department of Physiology, University of Louisville School of Medicine, Louisville, Kentucky 40202, USA; 3 Department of Surgery, University of Louisville School of Medicine, Louisville, KY 40202, USA; 4 Regenerative Bioscience Center, University of Georgia, Athens, Georgia 30602, USA; 5 Department of Animal and Dairy Sciences, University of Georgia, Athens, GA 30206, USA; 6 Georgetown College, Georgetown, KY 40324, USA; 7 Department of Physiology, Anatomy, and Genetics, University of Oxford, Oxford OX1 3QX, UK

## Abstract

Acquiring sufficient amounts of high-quality cells remains an impediment to cell-based therapies. Induced pluripotent stem cells (iPSC) may be an unparalleled source, but autologous iPSC likely retain deficiencies requiring correction. We present a strategy for restoring physiological function in genetically deficient iPSC utilizing the low-density lipoprotein receptor (LDLR) deficiency Familial Hypercholesterolemia (FH) as our model. FH fibroblasts were reprogrammed into iPSC using synthetic modified mRNA. FH-iPSC exhibited pluripotency and differentiated toward a hepatic lineage. To restore LDLR endocytosis, FH-iPSC were transfected with a 31 kb plasmid (*pEHZ-LDLR-LDLR*) containing a wild-type *LDLR* (FH-iPSC-LDLR) controlled by 10 kb of upstream genomic DNA as well as Epstein-Barr sequences (EBNA1 and *oriP*) for episomal retention and replication. After six months of selective culture, *pEHZ-LDLR-LDLR* was recovered from FH-iPSC-LDLR and transfected into *Ldlr*-deficient CHO-a7 cells, which then exhibited feedback-controlled LDLR-mediated endocytosis. To quantify endocytosis, FH-iPSC ± LDLR were differentiated into mesenchymal cells (MC), pretreated with excess free sterols, Lovastatin, or ethanol (control), and exposed to DiI-LDL. FH-MC-LDLR demonstrated a physiological response, with virtually no DiI-LDL internalization with excess sterols and an ~2-fold increase in DiI-LDL internalization by Lovastatin compared to FH-MC. These findings demonstrate the feasibility of functionalizing genetically deficient iPSC using episomal plasmids to deliver physiologically responsive transgenes.

Induced pluripotent stem cells (iPSC) derived from patients are a unique, potential alternative for replacing diseased tissue because of their virtually unlimited supply, differentiation potential, and tolerance by the immune system^1^. However, in the context of treating genetic disorders, autologous iPSC are challenged by their genetic homology with patient DNA, as they may retain functional deficiencies. Such shortcomings must be addressed before these cells can attain broad therapeutic value.

Familial Hypercholesterolemia (FH) is one example of a monogenic autosomal dominant disorder that affects low-density lipoprotein cholesterol (LDL) metabolism^2^. In FH, LDL receptor (LDLR) deficiency (attributed to at least one of 1200 documented mutations^3^,^4^) impedes receptor-mediated endocytosis of LDL, resulting in pathologically elevated serum LDL levels. Consequently, the formation of atherosclerotic plaques and cardiovascular disease are both accelerated. Because healthy liver hepatocytes contain the body’s highest density of functional LDLR^5^, liver transplant is regarded as the only cure for FH. Yet, the number of available livers is far outstripped by significant demand^6^, while long-term transplant issues such as immunomodulation and transplant rejection remain key hurdles. Further, liver hepatocytes culture poorly^7^, complicating efforts such as banking and expansion. For such reasons, generating hepatocytes from patient-specific iPSC (FH-iPSC) may be a possible therapeutic alternative.

We used FH as a model system for interrogating the LDLR functionalization of FH-iPSC. We restored FH-iPSC LDLR-mediated endocytosis using a novel 31 kb plasmid containing (a) wild-type *LDLR* physiologically controlled by 10 kb upstream genomic regulatory control sequence and (b) the minimum number of required Epstein-Barr Virus (EBV) replication and retention sequences (Epstein-Barr Nuclear Association 1 (EBNA1) and origin of plasmid replication (*oriP*))^8^. This large plasmid was transfected into iPSC via electroporation and retained as a stable episome. FH-iPSC were differentiated into hepatocyte-like cells (FH-HLC) and mesenchymal cells (FH-MC) to investigate LDL internalization. Transfected FH-iPSC derivatives demonstrated a significant, physiologically sensitive restoration of LDLR-mediated endocytosis compared to non-transfected controls. The technologies and methods discussed herein represent a unique approach for functionalizing genetically deficient iPSC using episomal plasmids that contain genomic transgene control sequences.

## Results

### FH-iPSC generation from FH fibroblasts

Skin fibroblasts from a patient with clinically homozygous FH (Coriell Cell Repositories; #GM01355) were examined to verify their inherent LDLR dysfunction using an LDL internalization assay (Supplemental Fig. S1). After pretreatment with Lovastatin, excess free sterols, and ethanol (vehicle control) in lipoprotein-deficient serum overnight, the cells were exposed to DiI-LDL the following day and found to be impaired in their ability to internalize LDL when compared against wild-type IMR90 fetal lung fibroblasts. The FH fibroblasts were then reprogrammed into FH-iPSC using transient, synthetic modified RNA (i.e. modRNA^9^; encoding *OCT4, SOX2, KLF4, c-MYC,* and *LIN28*^10^; Supplemental Results and Supplemental Figure S2), with a calculated efficiency of 0.012%. The FH-iPSC pluripotent potential was assessed via *in vitro* spontaneous differentiation and *in vivo* teratoma formation, with both assays demonstrating formation of the three germ layers (endoderm, mesoderm, and ectoderm). Karyotyping (Supplemental Figure S3) and DNA fingerprinting (not shown) (a) revealed no reprogramming-attributed chromosomal abnormalities and (b) demonstrated homology between the FH-iPSC and their source fibroblasts. These results demonstrate that the GM01355 FH fibroblasts were successfully reprogrammed to into pluripotent FH-iPSC.

### FH-iPSC differentiation towards a hepatic lineage

**Figure 1:**
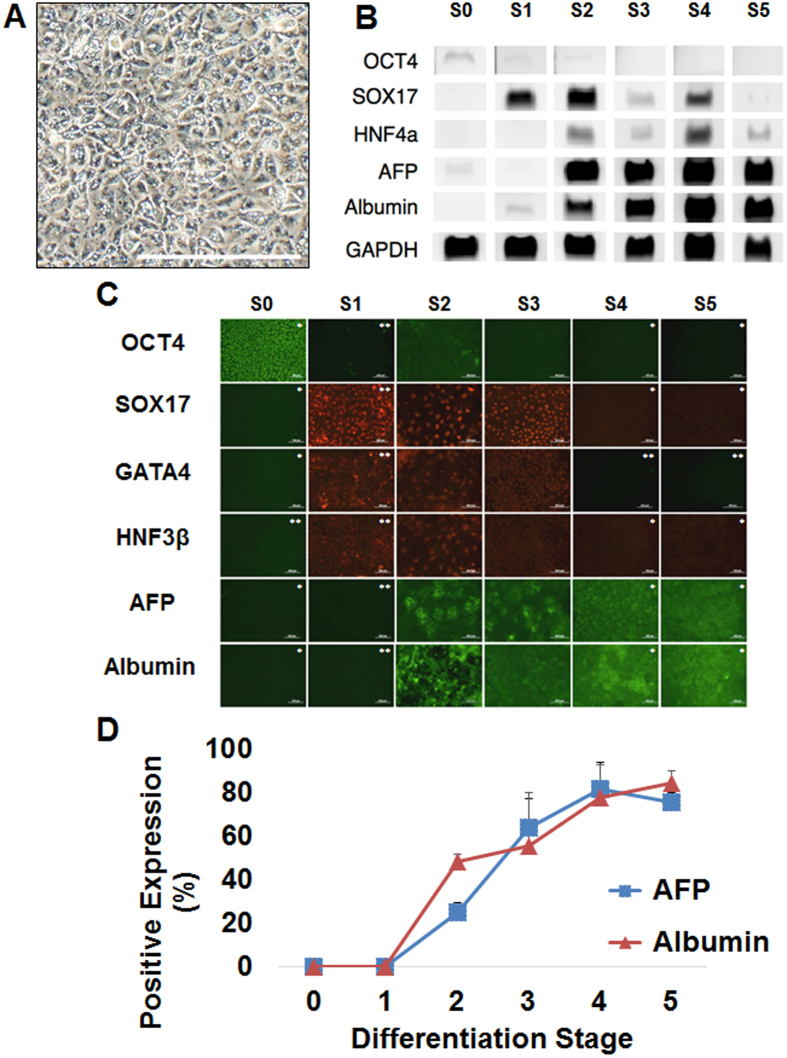
FH-iPSC Hepatic Differentiation. (**A**) Phase microscopy demonstrates hepatocyte-like cell (HLC) morphology at the end of the five-stage differentiation process (Scale bar = 500 μm). (**B**) Representative gel electrophoresis image of PCR transcripts at the end of each differentiation stage. GAPDH was used as a loading control. (**C**) Immunocytochemistry at the end of each differentiation stage demonstrates a progressive shift in protein expression towards a hepatic phenotype (Scale bars = 50 μm, *100 μm, and **200 μm; images of varying magnifications were chosen for clarity). (**D**) Quantification of AFP (blue)- and Albumin (red)-positive cells as a fraction of the total number of cells (DAPI), shown for each differentiation stage as mean ± S.E.M.

FH patients benefit from a variety of pharmacological and biomechanical interventions principally targeting liver hepatocytes. Thus, we wanted to determine if FH-iPSC could differentiate towards a hepatic lineage using a modified version of an established five-stage hepatic differentiation protocol that mimics key developmental embryologic cues (Fig. 1A)^11^. At the end of each stage, we conducted RT-PCR (Fig. 1B) and immunocytochemistry (Fig. 1C) to follow transcript and protein expression, respectively, throughout the spectrum of HLC derivation. Being undifferentiated, the FH-iPSC at Stage 0 expressed OCT4 transcripts and protein that rapidly decreased to undetectable levels in the subsequent stages. In stages 1 and 2, marked cell-specification into definitive endoderm and hepatic-defined lineages was evident from the nuclear expression of SOX17, HNF3β/4α, and GATA4. Starting in Stage 2, cells were positive for the hepatic markers Alpha-fetoprotein (AFP) and Albumin, which were initially observed as cytoplasmic clumps before attaining a more uniform distribution by Stages 3 and 4. By the end of Stage 5, AFP and Albumin transcript and protein expression predominated, indicating the generation of FH-HLC. Semi-quantitative PCR densitometry conducted at the end of each stage depicted a progressive change in gene expression, from pluripotence to markers associated with the hepatic lineage (Supplemental Fig. S4). The number of cells expressing AFP and Albumin at each stage was calculated as a percentage of all DAPI-labeled cells (Fig. 1D; mean ± S.E.M.). We noticed the following pattern of AFP expression over the course of the differentiation – Stage 0: 0%, Stage 1: 0%, Stage 2: 24.74 ± 4.73%, Stage 3: 63.84 ± 13.25%, Stage 4: 81.83 ± 11.99%, and Stage 5: 75.71 ± 4.08%. We witnessed a similar pattern of Albumin expression as well – Stage 0: 0%, Stage 1: 0%, Stage 2: 48.49 ± 3.31%, Stage 3: 55.3 ± 24.69%, Stage 4: 78.03 ± 14.92%, and Stage 5: 84.26 ± 5.73%. Thus, with respect to differentiation efficiency, most of the cells were positive for the two primary hepatic markers by the end of Stage 5 differentiation.

**Figure 2:**
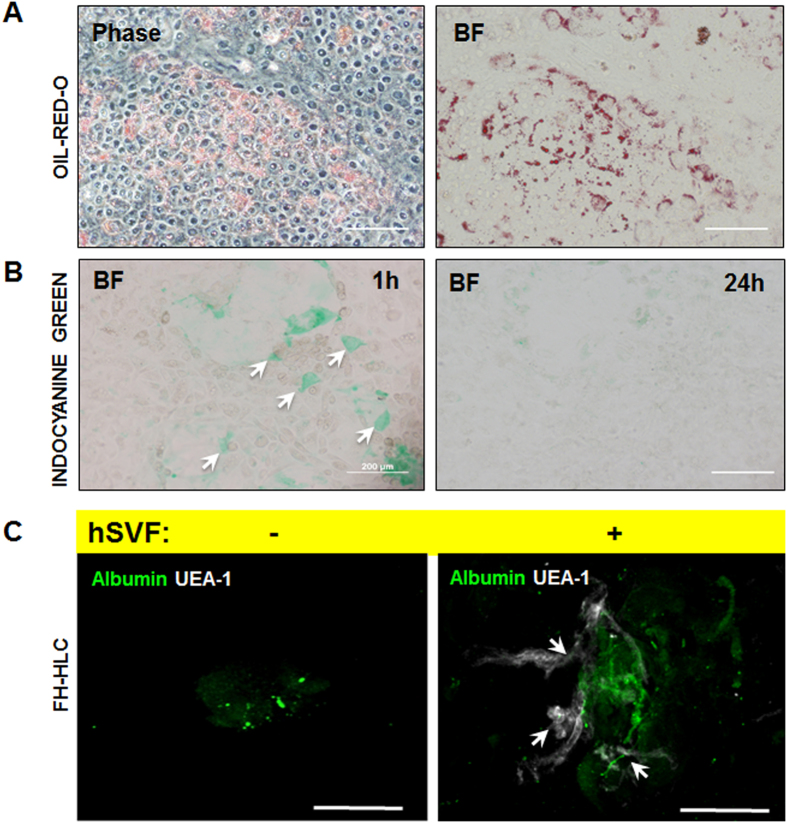
FH-HLC Functional Assays. (**A**) FH-HLC were stained with Oil-Red-O to visualize lipid accumulation with phase and bright field (BF) microscopy. (**B**) FH-HLC cellular uptake (white arrows) of Indocyanine Green (ICG) was imaged with BF microscopy after 1 hour of treatment. The same site was also assessed for ICG clearance 24 hours later. (Scale bar = 200 μm). **(C)** FH-HLC demonstrate survival and engraftment *in vivo* in Rag1^−/−^ × LDLR^−/−^ double-KO mice when implanted with adipose-derived stromal and vascular support cells (white arrows indicate network formation). FH-HLC without stromal support failed to survive or express Albumin after the two-week implant period. (Scale bar = 50 μm).

During each round of FH-HLC production, we noticed the presence of differentiating cells that took on an increasingly granular, vacuolar appearance (especially present at Stage 3) that looked lipid-like – an observation consistent with that of Fattahi and colleagues^12^. To determine if these vacuoles were in fact accumulating lipids, we subjected the FH-HLC to Oil-Red-O staining and subsequently confirmed the presence of numerous lipid droplets (Fig. 2A), suggesting that these cells are not impaired in their ability to store lipids, but perhaps only in their ability to bind and internalize LDL. This corresponds with what is seen clinically, as FH patients often exhibit normal liver function tests^13^. To determine if FH-HLC demonstrated any outward sign of maturation, the cells were treated with Indocyanine green (ICG), a clinically relevant dye that is metabolized and cleared by specific mature cell types including hepatocytes^14^. Though ICG was cleared, this observation was a relatively rare event, suggesting that the differentiation protocol yielded a predominantly immature hepatic population (Fig. 2B). Still, these results indicate that FH-iPSC can differentiate down a hepatic lineage into FH-HLC.

We then asked if FH-HLC could engraft *in vivo* (Fig. 2C). We previously demonstrated the utility of adipose-derived stromal vascular fraction cells (SVF) in providing vital stromal and vascular support^15^ to implanted parenchymal hepatocytes within a liver-tissue mimic^16^. Consequently, we combined human SVF with FH-HLC in collagen-I constructs and implanted them subcutaneously in Rag1^−/−^ × LDLR^−/−^ double-knockout mice for 2 weeks. We labeled the explanted constructs with Albumin-488 and UEA-1-Cy5 (a lectin that binds to human endothelium). Fluorescence confocal microscopy showed that FH-HLC had engrafted and expressed Albumin in the presence of SVF, whereas FH-HLC did not survive in the absence of SVF. Together, these findings suggest that FH-iPSC can differentiate down a hepatic lineage and, given the proper stromal and vascular support, survive and engraft *in vivo*.

### FH-iPSC transfection with *pEHZ-LDLR-LDLR*

FH-iPSC retain the same genetic dysfunction as their source patient fibroblasts and therefore offer limited therapeutic value with regards to restoring LDLR-mediated endocytosis. Since constitutive *LDLR* transgene expression for treating FH has not yet achieved a cure^17^,^18^ and may even be cytotoxic^19^,^20^ we elected to use the plasmid *pEHZ-LDLR-LDLR* to impart physiological responsiveness by 10 kb of up-stream genomic control sequences^8^,^21^,^22^.

We initially confirmed *pEHZ-LDLR-LDLR* physiological restoration of receptor-mediated endocytosis in the *Ldlr*-deficient cell line CHO-a7 (Supplemental Results and Supplemental Figure S5)^8^,^23^. Because of the plasmid’s large 31 kb size, we utilized electroporation transfection^24^ and tested a wide range of parameters using both decaying exponential and square-wave electroporators (parameters listed in Tables 1 and 2).

Using a BTX 630ECM decaying exponential electroporator with a 4 mm cuvette^24^, we found that settings achieving time constants (τ) between 16 and 18 ms were optimal for achieving iPSC survival and transfection (Table 1). Smaller τ were associated with increased iPSC survival but inadequate plasmid delivery, while τ greater than 18 ms resulted in cell death. We also tested the effects of temperature and buffer on electroporation success. For the conditions tested, pre-chilling all components was critical. Moreover, we found no difference with using either phosphate-buffered saline (PBS) or a commercially available electroporation buffer.

We then tested the NEPA21 square-wave electroporator using a range of parameters for plasmid delivery to human pluripotent stem cells. From the range of settings tested, a voltage of 300 V and pulse length time of 0.5 ms facilitated iPSC survival and successful delivery of the 31 kb plasmid (Table 2). We then conducted all square-wave electroporations at room temperature in Opti-MEM media. 48 hours after electroporation, Hygromycin-B selection was initiated at 10 μg/ml for at least 5 days, followed by 0.5–1 μg/ml thereafter as a maintenance dose.

### *pEHZ-LDLR-LDLR* retention and functional restoration

**Figure 3:**
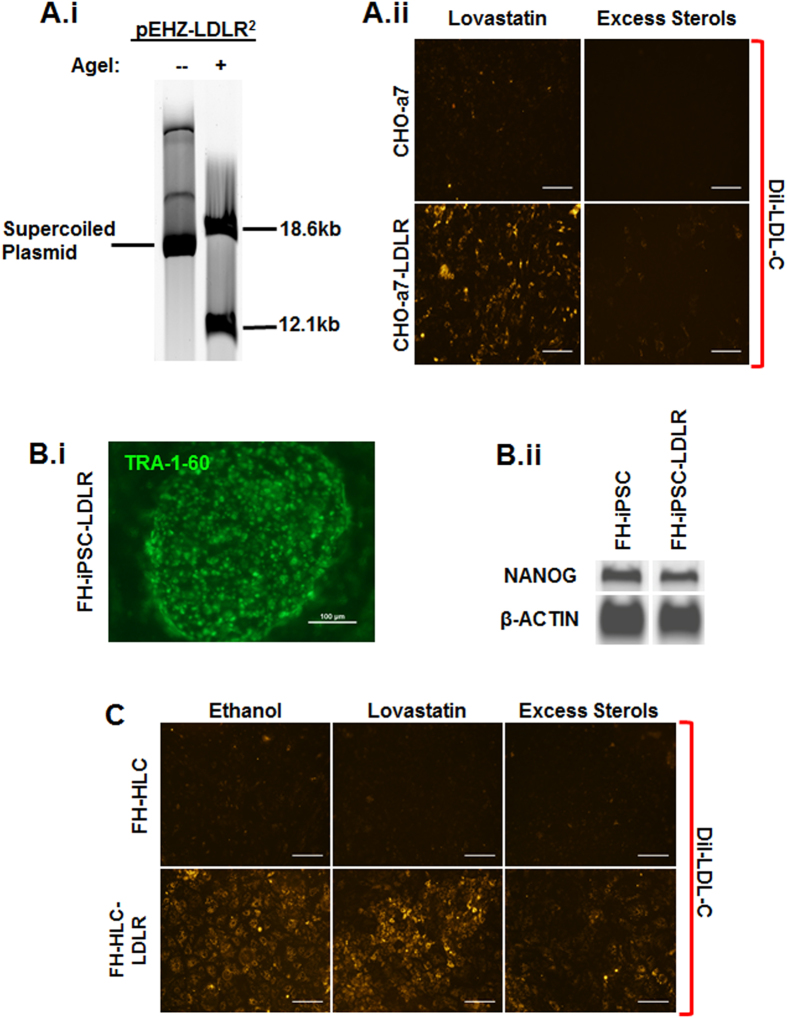
Extrachromosomal Plasmid Characterization. (**A.i)** *pEHZ-LDLR-LDLR* episome was recovered from FH-iPSC-LDLR and cleaves into expected fragment sizes after digestion by AgeI-HF restriction enzyme. (**A.ii**) Recovered plasmid transfected into *Ldlr-*deficient CHO-a7 demonstrates restored physiologic receptor-mediated endocytosis via DiI-LDL internalization. (**B.i**) FH-iPSC-LDLR colonies cultured for over 12 months still exhibit iPSC-characteristic well-defined borders and express TRA-1–60–488 via live-labeling (Scale bar = 100 μm). (**B.ii**) Pluripotence of long-term cultured FH-iPSC ± LDLR was verified by PCR, which demonstrated NANOG expression. β-actin was used as a loading control. (**C**) FH-HLC-LDLR displayed differential physiologic sensitivity, with highest DiI-LDL internalization with exposure to Lovastatin and almost complete internalization abrogation with exposure to excess sterols. Little internalization of DiI-LDL was detected in FH-HLC. Ethanol was used as a vehicle control (Scale bar = 200 μm).

*pEHZ-LDLR-LDLR* is stably maintained as an episome and does not integrate into the host genome^8^. After culturing FH-iPSC-LDLR under continuous Hygromycin-B selection^25^ for over 6 months, we subjected the cells to alkaline lysis and recovered the plasmid via ethanol precipitation. The isolated plasmid was purified and digested with the restriction enzyme AgeI, yielding two appropriately sized fragments measuring 18.6 kb and 12.1 kb (Fig. 3A.i). We then validated the functionality of the recovered plasmid by transfecting it into *Ldlr*-deficient CHO-a7 (CHO-a7-LDLR; Fig. 3A.ii). The recovered plasmid restored the physiological feedback control of LDLR-mediated endocytosis in the CHO-a7-LDLR. These findings collectively demonstrate that (a) *pEHZ-LDLR-LDLR* is retained as an episome in human iPSC and (b) its function is independent of genomic integration.

We next wanted to determine if *pEHZ-LDLR-LDLR* had an effect on the inherent pluripotence of our FH-iPSC. Therefore, we assessed expression of TRA-1–60 on transfected cells cultured for over a year under antibiotic selection pressure. Like their non-transfected FH-iPSC counterparts (Supplemental Fig. S2C), FH-iPSC-LDLR retained a robust expression of TRA-1–60 (Fig. 3B.i). Expression of NANOG in FH-iPSC ± LDLR was also confirmed by PCR (Fig. 3B.ii). These results suggest that transfection with the episomal plasmid *pEHZ-LDLR-LDLR* does not diminish iPSC pluripotence.

To determine if FH-iPSC-LDLR derivatives also retained the plasmid and displayed functional restoration of LDLR-mediated endocytosis, we differentiated FH-iPSC ± LDLR into FH-HLC±LDLR. The derived HLC were cultured with Lovastatin, excess free sterols, or ethanol vehicle control overnight in lipoprotein-deficient media prior to incubation with DiI-LDL (Fig. 3C). The FH-HLC demonstrated little internalization of DiI-LDL, indicating that without any transgene intervention, LDLR-mediated endocytosis of LDL remained dysfunctional. However, FH-HLC-LDLR exhibited markedly elevated LDL endocytosis that demonstrated the same physiological sensitivity to peripherally applied statin, sterols, or vehicle control as seen with the CHO-a7-LDLR (Supplemental Fig. S5). These findings indicate that *pEHZ-LDLR-LDLR* effectively restores physiological feedback control of LDLR activity in FH-iPSC derivatives.

### Restored receptor-mediated LDL endocytosis quantification

**Figure 4:**
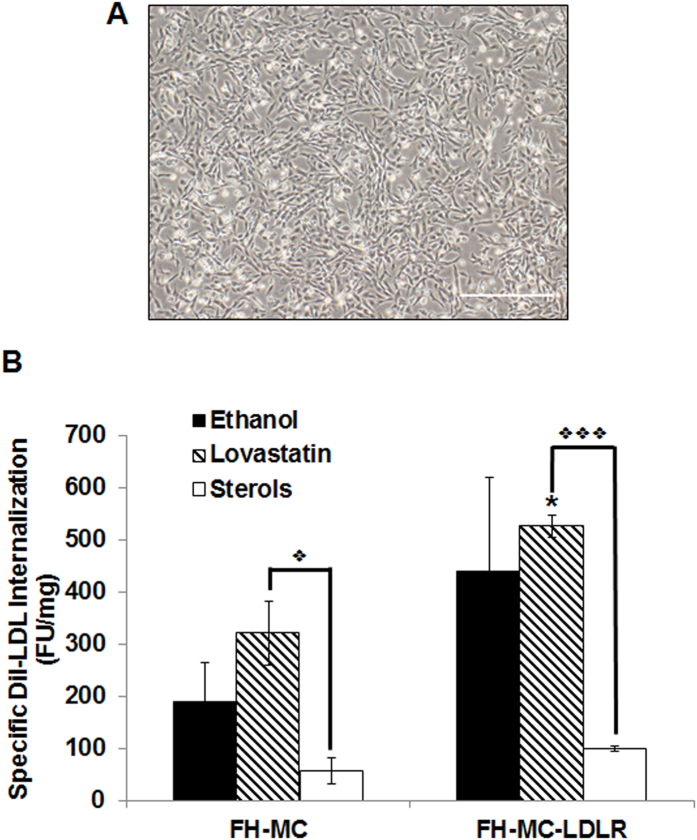
Quantification of LDL Receptor-Mediated Restoration. (**A**) Phase microscopy image of transfected FH-iPSC-derived mesenchymal cells (FH-MC±LDLR) (Scale bar = 500 μm) depicts a fibroblastic monolayer suitable for quantification studies. (**B**) Quantification of specific DiI-LDL internalization by FH-MC displayed impaired LDL internalization. Transfected FH-MC-LDLR demonstrated significantly greater ability to internalize DiI-LDL. Bars are depicted as means ± S.E.M., and these graphs characterize experiments conducted in triplicate (*,
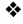
**  **= *p *< 0.05 and 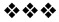 = *p *< 0.001; * = significance between cell types and similar treatments, while 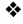 = significance within a cell type).

To facilitate the quantification of *pEHZ-LDLR-LDLR-*driven specific LDL internalization, we differentiated FH-iPSC ± LDLR into mesenchymal cells (FH-MC±LDLR; Fig. 4A) utilizing an established protocol from our lab^26^. These cells grow in a monolayer and are contact-inhibited, allowing quantification of specific LDL receptor-mediated endocytosis. FH-MC and FH-MC-LDLR were cultured in lipoprotein-deficient serum media supplemented with Lovastatin, excess sterols or ethanol control before exposure to DiI-LDL (Fig. 4B). The ethanol control yielded appreciable, though not significant (*p *= 0.27), differences in internalization between the two groups (FH-MC: 188.57 ± 74.33 vs. FH-MC-LDLR: 438.82 ± 180.57 fluorescence units/mg protein (FU/mg); mean ± SEM) reflecting the cellular lipid requirement due to the lipoprotein-deficient media environment. This difference was amplified when the cells were exposed to the additional stimulus of Lovastatin. Transgene-expressing cells displayed a statistically significant increase (*p *= 0.035) in specific internalization versus their non-transfected counterparts (FH-MC: 321 ± 61.7 FU/mg vs. FH-MC-LDLR: 526.15 ± 21.18 FU/mg). As expected, free sterols suppressed DiI-LDL internalization to a statistically equivalent level regardless of transgene presence (FH-MC: 56.90 ± 25.93 vs. FH-MC-LDLR: 99.15 ± 5.80 FU/mg, *p *= 0.19) since free sterol internalization is receptor independent^27^. Within the transfected FH-MC-LDLR population, the suppression elicited by free sterols was extremely significant when compared to Lovastatin (*p *= 0.00004) and significant with regards to the ethanol control (*p *= 0.02). Presenting these differences another way, transfected FH-MC-LDLR demonstrated 1.63- and 2.33-fold increases in specific DiI-LDL internalization over FH-MC in the presence of Lovastatin and ethanol, respectively. These findings suggest that the *pEHZ-LDLR-LDLR* plasmid functionally restores LDL receptor-mediated endocytosis without direct genetic manipulation in FH-iPSC-derived progeny.

## Discussion

Here, we present a proof-of-concept strategy with broad implications for generating and functionally restoring genetically deficient iPSC utilizing transient reprogramming and non-viral, episomal vectors with genomic transgene control, respectively. The significant findings of this project are that (a) an episomal transgene plasmid can be maintained in an extra-chromosomal position within iPSC, (b) the plasmid does not diminish iPSC pluripotency, and (c) genomic control of plasmid transgene expression can restore physiologic functionality to genetically deficient cells.

We used modRNA to reprogram FH-fibroblasts into FH-iPSC. modRNA is reported to yield one of the highest reprogramming efficiencies, approaching 2% in normal fibroblasts^28^. The fact that our efficiency was considerably lower than published figures could potentially be attributed to the LDLR deficiency of our source FH fibroblasts, however other reports describing the generation of FH-iPSC using viral vectors do not report reprogramming efficiencies^12^,^29^,^30^. As such, we may simply be observing experimental variation, and practically, our calculated efficiency is not a significant concern given the highly proliferative nature of iPSC.

With regards to genetic correction, attempts to resolve LDLR deficiency via gene therapy approaches have primarily relied on viral vectors^31^,^32^,^33^,^34^. Published approaches have highlighted the delivery of infectious viral particles via the portal vein to target the liver. In animal models, this has resulted in two possible outcomes: (a) a reduction in circulating LDL that was significant, but transient^31^, or (b) a total reduction in LDL that did not achieve normal cholesterol levels due to possible plasmid loss^35^, transgene silencing^36^, host immune detection of transduced cells^32^, or unknown hepatocyte transduction efficiency^31^,^33^. Commonly used viral vectors have a relatively small (less than 10 kb) insert capacity, thereby limiting the options for controlling transgene expression^37^. Heeren and colleagues reported that the constitutive LDLR expression controlled by either Rous Sarcoma Virus or Cytomegalovirus (CMV) promoters resulted in cytotoxicity due to excessive lipid internalization^19^,^20^. Kozarsky also reported lipid accumulation in the livers of Watanabe heritable hyperlipidemia (WHHL) rabbits treated with CMV-driven *LDLR* in E1-deleted adenovirus^38^. Recent reports of *LDLR* gene therapy with constitutive transgene expression driven by hepatocyte-specific promoters (such as α1-antitrypsin or thyroid binding globulin) did not detect cytotoxic lipid accumulations^31^,^34^. Thus, controlling *LDLR* expression with hepatocyte-specific promoters may provide some unidentified mechanism for protecting against lipid accumulation^39^. However, considering the highly regulated expression of the normal LDLR, constitutive LDLR expression may not be without long-term detrimental consequences.

Episomal plasmids containing genomic DNA expression control sequences are an alternative approach to addressing the *LDLR* genetic deficiency. The inclusion of genomic sequences allows for precise physiological control of the transgene and enhances long-term *LDLR* expression^24^. The plasmid used here, *pEHZ-LDLR-LDLR*, is maintained as an episome by the inclusion of the EBV retention and replication sequences EBNA1 and *oriP*^8^. The EBNA1 *trans* regulatory protein is the only EBV protein required for retained episomal function^40^. Though these sequences are virus-derived, host immune cells do not detect EBNA1 (as opposed to other EBV native proteins)^41^, thus reducing the host’s potential immunogenic response to the plasmid.

As previously mentioned, *LDLR* expression is tightly controlled by a feedback mechanism within the cell^42^. Hibbitt and colleagues demonstrated that the *pEHZ-LDLR-LDLR* plasmid restored receptor-mediated endocytosis in FH fibroblasts and persisted long-term in non-dividing hepatocytes *in vivo*^8^, although the transfection efficiency was unclear. Here, we demonstrate that *pEHZ-LDLR-LDLR* restores physiologically sensitive receptor-mediated endocytosis in FH-iPSC and that this function is retained after differentiation into various progeny.

We also demonstrate the effectiveness of large plasmid transfection into reprogrammed iPSC. Although transfection of the source FH fibroblasts before reprogramming is an option, it is unknown if (potentially multiple copies of) a large 31 kb episomal plasmid would affect reprogramming or if long-term plasmid retention would be compromised. Directly transfecting the FH-iPSC derivatives was also an option, but the cells would still require the time and culture for antibiotic selection, both of which are problematic with hepatocytes^7^. Though electroporation is a relatively inefficient means of plasmid delivery, in principle, only one transfected cell is required to generate a theoretically unlimited resource for cells with functionally restored feedback control of LDLR endocytosis. As a future FH therapeutic, if a sufficient quantity of cells carrying the genomic DNA-controlled transgene could be delivered, then host homeostatic control of LDL metabolism could be restored without the need for constitutive *LDLR* expression.

A major issue to address with our approach is plasmid retention, a common problem with using episomal viral vectors for direct gene therapy to the liver^43^. EBV-based plasmids replicate once per cell cycle^44^ and, without antibiotic selection, are lost at a rate of 2 to 5% per cell division^21^. Therefore, using iPSC as a therapeutic cell source may require continuous selection until a stable phenotype (such as hepatocytes) can be derived. To that end, two approaches could be used to achieve this goal: (a) differentiate FH-iPSC-LDLR *in vitro* with antibiotic selection until a minimally proliferative state is reached prior to implantation, or (b) generate a liver-tissue mimic with stromal and vascular support that facilitates selection and phenotype stabilization prior to implantation.

Though our approach specifically targets the LDLR dysfunction in FH, a strategy using episomes with genomic control of transgenes can be tailored to other biological conditions or clinical deficiency with potential for amelioration by iPSC intervention, further enhancing its clinical potential in tissue engineering applications.

## Methods

### Ethics Statement

All animal procedures were conducted in compliance with University of Louisville School of Medicine IACUC-approved protocols and NIH guidelines. All recombinant DNA work was approved by the University of Louisville Institutional Biosafety Committee and performed in accordance with NIH guidelines.

### Hepatocyte-Like Cell Production

FH-iPSC were plated to confluence in Matrigel-pretreated wells of a 6-well tissue culture plate or 6 Permanox chamber slides (Sigma-Aldrich). Each well of the 6-well plate (used for PCR analysis) and each Permanox slide (used for immunocytochemistry) corresponded with one of the six stages of differentiation (including Stage 0, which represented untreated iPSC). To generate HLC, we used a modified version of the protocols and medias proposed by Song *et al.*^11^. In lieu of Song’s Stage 1 media, we utilized the StemDiff definitive endoderm kit (Stem Cell Technologies) per the manufacturer’s instructions. Media changes were performed daily and cells were characterized by the end of Stage 5.

### Imaging of Hepatocyte-Like Cell LDLR-Mediated Endocytosis

FH-HLC derived from transfected FH-iPSC (FH-HLC-LDLR) were starved overnight in 5% lipoprotein-deficient serum supplemented with 2 μM of Lovastatin, excess sterols (12 μg/ml cholesterol and 0.6 μg/ml 25-hydroxysterol), or ethanol (vehicle control). The following day, the starvation media was replaced with 5 μg/mL DiI-LDL in DMEM-HG for 4.5 hours. Cells were thoroughly washed with di-cationic PBS prior to imaging via an Olympus IX81 fluorescence microscope.

### Plasmid Rescue and Restriction Digest

Plasmid rescue was performed on transfected FH-iPSC-LDLR using methods described by Lufino and colleagues^25^. The rescued plasmid was characterized using AgeI-HF restriction enzyme (New England BioLabs) per manufacturer instructions. Restriction products were run on a 0.4% agarose gel at 20 V for 16 hours. Gels were analyzed via a Typhoon 9400 variable mode imager (GE Healthcare Bio-Sciences). After confirming plasmid isolation, the rescued plasmid was also re-transfected into *Ldlr-*deficient CHO-a7 cells (generously provided by Prof. Monty Krieger, Massachusetts Institute of Technology) via Lipofectamine 2000 to demonstrate non-integration and preservation of plasmid functionality after rescue.

### FH Mesenchymal Cell Generation and Culture

Mesenchymal cells were generated as previously described^26^. FH-iPSC±LDLR were passaged onto Matrigel-coated plates using Gentle Cell Dissociation Buffer (Stem Cell Technologies) and introduced to 100% mTeSR1 in a graded manner. One day after passaging, transfected cultures received 1 μg/ml Hygromycin B to maintain selective pressure. Once confluent and in 100% mTeSR1, the media was changed to EGM2-MV (Lonza) for 20 days for differentiation into FH-MC. Once fully differentiated, FH-MC were passaged and used in LDL internalization assays.

### Quantification of *pEHZ-LDLR-LDLR* Activity

FH-MC±LDLR were plated in gelatin-coated wells of a 24-well tissue culture plate. We utilized an established protocol^45^, but used fluorescent DiI-LDL (Alfa Aesar) in place of ^125^Iodinated-LDL. Specific receptor-mediated internalization was determined by subtracting the internalization of [DiI-LDL + 50-fold excess of unlabeled LDL (Alfa Aesar)] from the internalization quantified with DiI-LDL alone. Cells were then lysed with 250 μl 0.1 N NaOH (Ricca Chemical Company). Fluorescence was quantified using a 96-well plate reader (BioTek), with the excitation and emission set to 514 nm and 550 nm, respectively. Fluorescence units were normalized to total protein content, which was determined via a DC Protein Assay kit (Bio-Rad).

### Statistical Analysis

Experiments were conducted in triplicate with duplicated data points per experiment. Values are presented as means ± S.E.M. Significance was determined using a Student’s *t*-test and confirmed via one-way ANOVA using SigmaPlot (Systat Software).

Additional procedures can be found in the Supplemental Methods.

## Additional Information

**How to cite this article**: Ramakrishnan, V. M. *et al.* Restoration of Physiologically Responsive Low-Density Lipoprotein Receptor-Mediated Endocytosis in Genetically Deficient Induced Pluripotent Stem Cells. *Sci. Rep.* **5**, 13231; doi: 10.1038/srep13231 (2015).

## Supplementary Material

Supplementary Information

## Figures and Tables

**Table 1:** FH-iPSC decaying exponential electroporation transfection parameters.

Cells(×10^6^)	Plasmid(μg)	Voltage(V)	Capacitance(μF)	Resistance(Ω)	τ (ms)	Temp(°C)	Vol(μl)	Buffer	Rock InhibitorPretreatment	Electro.Survival	HygromycinSurvival
10	35	300	960	200	23.1	4	300	MEB	N	N	NA
10	35	320	250	0	8	4	300	MEB	N	Y	N
10	100	300	250	0	6.9	4	700	PBS	Y	Y	N
10	100	300	975	0	16.4	4	700	PBS	Y	Y	Y
10	100	300	975	200	23.1	RT	300	MEB	Y	N	NA
10	100	300	975	0	18	4	700	MEB	Y	Y	Y
3	25	300	975	0	31.7	4	350	MEB	Y	N	NA
10	25	170	200	1000	9	4	300	PBS	N	Y	N
10	50	170	200	1000	10.5	4	300	PBS	N	Y	N
10	100	170	200	1000	11.2	4	300	PBS	N	Y	N
10	25	170	250	1000	12.6	4	300	PBS	N	Y	N
10	50	170	250	1000	12.7	4	300	PBS	N	Y	N
10	100	170	250	1000	13.4	4	300	PBS	N	Y	N
10	35	300	960	200	23.1	RT	300	MEB	N	N	NA
10	35	320	250	0	8	RT	300	MEB	N	Y	N

We used the BTX 630 with a 4 mm Gap Cuvette. Greyed rows indicate the parameters that yielded surviving, transfected FH-iPSC with *pEHZ-LDLR-LDLR.*

**Table 2:**
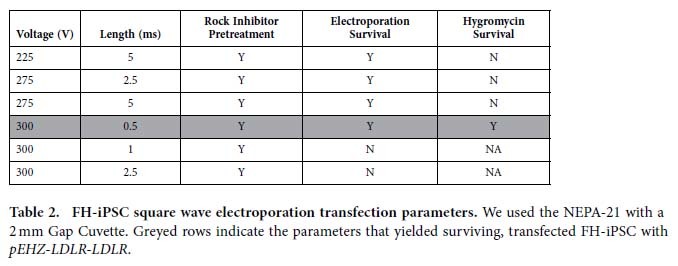
FH-iPSC square wave electroporation transfection parameters.
