# Supplementary material for: Restoration of Physiologically Responsive Low-Density Lipoprotein Receptor-Mediated Endocytosis in Genetically Deficient Induced Pluripotent Stem Cells
Source: Sci Rep. 2015 Aug 26;5:13231. doi: 10.1038/srep13231 (PMC4549683; doi:10.1038/srep13231)
Supplement: Supplementary Information [file srep13231-s1.doc]

**Restoration of Physiologically Responsive Low-Density Lipoprotein Receptor-Mediated Endocytosis in Genetically Deficient Induced Pluripotent Stem Cells**

Venkat M. Ramakrishnan*, Jeong Yeh Yang*, Kevin T. Tien, Thomas R. McKinley, Braden R. Bocard, John G. Maijub, Patrick O. Burchell, Stuart K. Williams, Marvin E. Morris, James B. Hoying, Richard Wade-Martins, Franklin D. West, and Nolan L. Boyd

**SUPPLEMENTAL DATA**

**SUPPLEMENTAL RESULTS**

**Non-viral synthetic modified mRNA reprogramming to generate FH-iPSC.** Because concerns with iPSC generation include (a) genomic integration of exogenous reprogramming factors and (b) unwanted spontaneous transcriptional activation post genomic integration, we sought to utilize mRNA-based reprogramming technologies. FH patient fibroblasts were obtained from Coriell Cell Repositories (#GM01355; Supplemental Fig. S2A) and transfected with synthetic modified mRNA^1^ (modRNA) (encoding *OCT4, SOX2, KLF4, cMYC* and *LIN28^2^*). Within 20 days of continuous transfection, the fibroblasts gradually attained a compacted morphology broadly characteristic of iPSC, with a calculated reprogramming efficiency of 0.012% (Supplemental Fig. S2B). Live-cell immunolabeling with the extracellular pluripotence marker TRA-1-60 (Supplemental Fig. S2C) suggested successful iPSC reprogramming.

Over the next several days, we observed colonies that possessed increasingly defined borders and cells with high nuclear-to-cytoplasmic ratios and prominent nucleoli. These cell clusters were positive for the intracellular pluripotent markers OCT4 (Supplemental Fig. S2D) and SOX2 (Supplemental Fig. S2E), which co-localized (Supplemental Fig. S2F), giving visual credence to the fact that the source fibroblasts were fully reprogrammed into iPSC^3^. Additionally, the extracellular surface pluripotent markers SSEA4 (Supplemental Fig. S2G), TRA-1-60 (Supplemental Fig. S2H), and TRA-1-81 (Supplemental Fig. S2I) were also visualized. The expression of these intracellular and extracellular pluripotent markers coupled with the aforementioned hPSC-specific morphologic features suggested a successful development of FH patient-specific iPSC from source fibroblasts. To further characterize their lineage and normality, FH fibroblasts and FH-iPSC were subjected to karyotypic analysis (Supplemental Figs. S3A & B) and DNA fingerprinting (not shown), confirming genetic homology between the two cell populations. This validated our production of autologous iPSC and ruled out any experimentally induced genetic abnormalities or variations.

We next assessed FH-iPSC pluripotency by spontaneous differentiation *in vitro* and teratoma formation *in vivo*. After 20 days of culture without supplemental bFGF, cells expressed the extracellular markers β-III TUBULIN (ectoderm; Supplemental Fig. S2J), VIMENTIN (endoderm; Supplemental Fig. S2K) and α-SMOOTH MUSCLE ACTIN (α-SMA; mesoderm; Supplemental Fig. S2L). For *in vivo* testing, undifferentiated FH-iPSC were combined with Matrigel and injected into the right subcutaneous dorsum of NOD/SCID-γ^null^ immunocompromised mice, while the left dorsum was injected with Matrigel alone as a negative control. Injected FH-iPSC yielded teratomas with evidence of solid tissue development and displayed a vasculature that anastomosed with that of the host. Histologic assessment of the solid tissue showed clusters of neuroepithelial cells (ectoderm; Supplemental Fig. S2M), regions of cartilage and immature bone formation (mesoderm; Supplemental Fig. S2N), and organized ciliated columnar epithelium (endoderm; Supplemental Fig. S2O). Expectedly, the Matrigel negative control yielded no teratomas (data not shown). This data demonstrates the utility of transient, non-integrating mRNA reprogramming vector systems in generating pluripotent, patient-specific iPSC.

**Verification of *pEHZ-LDLR-LDLR* functionality.** To verify the functionality of *pEHZ-LDLR-LDLR*, we utilized CHO-a7, an *Ldlr*-deficient cell line^4,5^ (CHO-a7-LDLR) (Supplemental Figure S5). Transfected CHO-a7 were selected with Hygromycin-B then compared against wild-type CHO (CHO-WT) and non-transfected CHO-a7 on the basis of their ability to bind and internalize fluorescent DiI-LDL cholesterol. Each CHO-a7 was treated overnight with Lovastatin, excess free sterols, or ethanol (a vehicle control) in lipoprotein-deficient serum. Lovastatin is an HMG-CoA reductase inhibitor that blocks the rate-limiting step in cholesterol synthesis^5,6^. By impeding the cells’ endogenous production of LDL-C, Lovastatin facilitates exogenous cholesterol internalization via surface up-regulation of the LDLR, allowing the cell to still meet its metabolic requirements while having an overall therapeutic effect on serum cholesterol levels. Excess free sterols are membrane permeable. Consequently, they can be internalized by receptor-independent mechanisms that secondarily result in LDLR down-regulation^7^. Under such conditions, CHO-WT showed an expected pattern of maximum DiI-LDL internalization with Lovastatin treatment over the ethanol control, and almost complete abrogation of DiI-LDL uptake when pre-treated with excess free sterols. In contrast, CHO-a7 expectedly demonstrated virtually no DiI-LDL internalization regardless of the treatment condition. The transfected CHO-a7-LDLR exhibited DiI-LDL internalization that was appropriately sensitive to exogenous Lovastatin and demonstrated functional restoration of LDL receptor-mediated endocytosis. Exposure to excess sterols yielded a decrease in LDL-C internalization, while ethanol vehicle treatment yielded a slight increase. This behavior parallels that seen in *Ldlr*-competent CHO-WT under the same experimental conditions, validating the *LDLR* functionality and physiologic sensitivity of the episomal plasmid.

**SUPPLEMENTAL METHODS**

**General Cell Culture.** Mouse embryonic fibroblasts (MEF) were cultured in MEF media comprised of DMEM-HG, 2 mM L-glutamine, and 10% standard fetal bovine serum (FBS; Invitrogen). Chinese hamster ovary wild-type (CHO-WT) and *LDLR* deficient (CHO-a7) cells (generously provided by Prof. Monty Krieger, Massachusetts Institute of Technology) were cultured in media containing DMEM/F12 (Invitrogen), 2mM L-glutamine, and 10% standard FBS. All cells were maintained at 37˚C and 5% CO_2_ and passaged at confluence with 0.05% Trypsin-EDTA (Invitrogen).

**Parent Fibroblast Culture.** Dermal fibroblasts from an FH patient (GM01355; Coriell Cell Repositories) were cultured in FH growth medium consisting of MEM (Invitrogen), 15% HyClone™ defined FBS (Thermo Fisher Scientific), 2 mM L-glutamine, 0.1 mM Non-Essential Amino Acids (MNEAA), 50 units/ml penicillin and 50 μg/ml streptomycin (Invitrogen). WT IMR90 fibroblasts were cultured in MEF media as described above. All cells were maintained in standard cell culture conditions and passaged with 0.05% Trypsin-EDTA.

**iPSC Generation and Culture.** Reprogramming was performed in 6-well tissue culture dishes coated with 0.2% gelatin (Sigma-Aldrich). On Day -2, Mitomycin-C-inactivated human foreskin fibroblast feeder cells (Nuff; GlobalStem) were plated at a density of 2.5x10^5^ cells/well and cultured in Nuff growth media, comprised of DMEM-HG, 10% HyClone defined FBS, 1% GlutaMAX, 50 units/ml penicillin and 50 μg/ml streptomycin (Invitrogen). On Day -1, 2.5x10^4^ FH parent fibroblasts were seeded atop the Nuff feeder layer in FH growth medium. After overnight plating, fibroblasts were transfected daily for 20 days in Pluriton reprogramming medium supplemented with 200 ng/ml B18R interferon inhibitor (Stemgent), the mRNA cocktail, and Lipofectamine-RNAiMax Transfection Reagent (Invitrogen) per the manufacturers’ instructions. Fresh reprogramming medium was substituted after 4 hours of incubation. Pluripotent colonies (FH-iPSC; TRA-1-60^+^ via staining with StainAlive TRA-1-60 (Stemgent)) were identified and manually passaged from transfected cultures onto Mitomycin C-inactivated (Sigma-Aldrich) MEF every three days. FH-iPSC were cultured with human iPSC medium (DMEM/F12 supplemented with 20% Knockout Serum Replacement (KSR), 2 mM L-glutamine, 0.1 mM MNEAA (Invitrogen), 0.1 mM β-mercaptoethanol (Sigma-Aldrich) and 20 ng/ml fibroblast growth factor-2 (bFGF; Stemgent))^8^. For feeder-free culture, FH-iPSC were passaged onto hESC-Qualified Matrigel coated plates (BD Biosciences) and gradually adapted to mTeSR1 media (Stemcell Technologies) by serial transition from 20% KSR culture media over a 10-day period with daily media changes. Cells in feeder-free culture were used for parenchymal cell generation before the 10^th^ passage^9^.

**FH Cell Characterization.** DNA karyotyping and fingerprint analysis (Cell Line Genetics) was completed on FH skin fibroblasts and reprogrammed iPSC (P21) to ensure that both populations were karyotypically normal and maintained identical DNA fingerprints. To verify disease phenotype, FH fibroblasts were compared against wild-type IMR90 fetal lung fibroblasts on the basis of their ability to internalize LDL cholesterol (see Supplemental Fig. S1). FH-iPSC pluripotence was then assessed via *in vitro* spontaneous differentiation and *in vivo* teratoma assays. *In vitro*, FH-iPSC were plated on glass slides in differentiation medium (human iPSC medium without bFGF) and differentiated for 20 days. 50% media changes were performed every other day. After 20 days, we assessed the iPSC for pluripotent marker expression via immunocytochemistry. For *in vivo* teratoma generation, we utilized FH-iPSC that were passaged with 0.05% Trypsin-EDTA at least five times prior^10^. Growth-Factor Reduced Matrigel (BD Biosciences) was mixed with an FH-iPSC cell pellet to a final concentration of 2.5x10^4^ cells/μl; 200 μl was loaded into sterile 1ml syringes with 18G needles (BD Biosciences). Control syringes containing only Matrigel were also prepared. iPSC and controls were injected on opposing sides into the subcutaneous dorsum of isoflurane-anesthetized NOD/SCID-γ mice (Jackson Laboratories). Injected mixtures were allowed 15 minutes to polymerize before the cessation of anesthesia. At 7 weeks post injection, masses were palpable in the flanks of the animals; at 14 weeks, the teratomas were visualized *in situ*, excised with gross margins and processed for histological examination.

**Verification of Plasmid Functionality.** To verify plasmid functionality, *Ldlr*-deficient CHO-a7 cells were first plated to confluence in a 48-well tissue culture dish. Once confluent, CHO-a7 cells were transfected with a complex of plasmid and Lipofectamine 2000 (Invitrogen) in a 1 μg : 1 μl ratio in Opti-MEM (Invitrogen) per manufacturer instructions^11^. Cells were cultured overnight in standard culture conditions. The following day, transfection media was removed and replaced with standard CHO-culture media (described above). 24 hours later, selection for transfected CHO-a7 (CHO-a7-LDLR) took place via exposure to 750 μg/ml Hygromycin B in CHO culture media. Afterwards, cells were starved overnight in media comprised of DMEM-HG, 1X L-glutamine and 5% lipoprotein-deficient serum (Alfa Aesar). Starvation media was augmented with 2 μM of Lovastatin (EMD Millipore), excess sterols (12 μg/ml cholesterol and 0.6 μg/ml 25-hydroxysterol; Sigma-Aldrich), or ethanol (vehicle control; Sigma-Aldrich)^5^. The following day, the starvation media was removed and replaced with 5 μg/ml DiI-LDL (Alfa Aesar) in DMEM-HG for 4.5 hours, after which the cells were thoroughly washed with di-cationic PBS and imaged using an Olympus IX81 fluorescence microscope.

**FH-iPSC Electroporation and Selection.** We transfected FH-iPSC with the corrective plasmid using both decayed-exponential and square-wave electroporation modalities. For FH-iPSC used in decayed-exponential electroporation (conducted at 4˚C), cells grown in feeder-free culture were pretreated with 10 μM of Y-27632 (EMD Millipore) two hours prior to electroporation. The cells were treated with 1 mg/ml Collagenase Type IV (Invitrogen) for 10 minutes, resuspended in DMEM/F12, and counted. We utilized a minimum of 10^7^ cells per electroporation. Cells were pelleted and resuspended in 700 μl of ice-cold (4˚C) di-cationic PBS or ES-qualified electroporation buffer (MEB; EMD Millipore). 100 μg of plasmid DNA (per 10^7^ cells) was Q.S. to 50 μl with electroporation buffer. Cell and plasmid solutions were combined; the resulting 750μl volume was added to a pre-chilled (4˚C) 4mm gap cuvette (BTX) and gently mixed by flicking. Cells were electroporated (conditions are outlined in Table 1) using an ECM 630 Electro Cell Manipulator (BTX; generously provided by Dr. Scott Whittemore, University of Louisville). Electroporated cells were incubated on ice for 5 minutes and suspended in 10 ml DMEM/F12 before pelleting. The pellet was resuspended in mTeSR1 with 10 μM Y-27632 and plated into 3 Matrigel-coated wells of a 6-well tissue culture dish. For FH-iPSC used in square wave electroporation (carried out at room temperature), cells were pretreated with 10 μM Y-27632 2 hours prior to electroporation. The cells were harvested via a 10-minute application of 1 mg/ml Collagenase Type IV and washed twice with Opti-MEM. Cells were placed into microcentrifuge tubes at a ratio of 10^6^ : 90 μl Opti-MEM. Plasmid DNA was maintained in a separate tube at 1 μg/μl in TE Buffer (Ambion). Recipient 6-well tissue culture dishes were pre-loaded with mTeSR1 and 10 μM Y-27632 and kept at 37˚C. Prior to electroporation, 10 μl of plasmid was mixed with 90 μl of iPSC (total electroporation volume = 100 μl) and transferred to an electroporation cuvette (NepaGene). The cuvette was gently flicked to ensure adequate mixture. Cells were electroporated using a NEPA21 cell electroporator (NepaGene; generously provided by Dr. Michal Hetman, University of Louisville; parameters are outlined in Table 1). Transfected cells were directly transferred into each well of the 6-well tissue culture recipient plate. For both conditions, after electroporation, cells were cultured for 2 days in mTeSR1 before a 5-day selection with 10 μg/mL Hygromycin B and subsequent maintenance in 0.5 to 1 μg/mL Hygromycin B, indicating successful transfection. Media was changed daily.

**Hepatocyte-Like Cell General Functional Assessment.** Indocyanine green (ICG; Sigma-Aldrich; 5 mg/ml in sterile water) was used to measure overall cell metabolic activity. ICG and Stage 5 media were mixed per manufacturer’s instructions and applied to FH-HLC for 1 hour. FH-HLC were thoroughly washed with di-cationic PBS to remove residual dye before being imaged on an Olympus IX81 microscope via phase and bright field settings. FH-HLC were then incubated in Stage 5 media overnight before assessment 24 hours later using the same microscope settings. An Oil-Red-O assay was used to evaluate cell lipid accumulation. A different population of FH-HLC was treated with Carnoy’s fixative for 2 minutes at RT before being washed with distilled water. Working Oil-Red-O solution (Sigma-Aldrich) was prepared per the manufacturer’s instructions and applied to FH-HLC for 30 minutes at RT. Cells were thoroughly washed with distilled water and imaged on an Olympus IX81 microscope with phase and bright field settings.

**Transfected Hepatocyte-Like Cell Qualitative Imaging.** FH-HLC derived from transfected FH-iPSC (FH-HLC-LDLR) were grown in 3 wells of a 12-well tissue culture plate. Cells were starved overnight in 5% lipoprotein-deficient serum supplemented with 2 μM of Lovastatin, excess sterols (12 μg/ml cholesterol and 0.6 μg/ml 25-hydroxysterol), or ethanol (vehicle control). The following day, starvation media was removed and replaced with 5 μg/mL DiI-LDL in DMEM high glucose for 4.5 hours. Cells were thoroughly washed with di-cationic PBS prior to imaging via an Olympus IX81 fluorescence microscope.

**Hepatocyte-Like Cell *In Vivo* Assessment.** Human SVF (hSVF) was grown to confluence *in vitro* in a gelatin-coated T75 flask while FH-HLC were grown to confluence all the way through end of Stage 2. On the day of implantation, both hSVF and HLC were passaged using 0.05% Trypsin-EDTA. Constructs were prepared using 10^6^/ml HLC ± 10^6^/ml of SVF in a master mixture of 3 mg/ml Collagen I (comprised of Rat Tail Collagen I (BD Biosciences), 4X DMEM (Invitrogen; made from 10X powdered concentrate), Sterile Water, and 0.1N NaOH (Ricca Chemical Company)). 250 µl of the cellular mixture was pipetted into each required well of a 48-well plate and left at 37˚C to polymerize for 30 minutes. Afterwards, the constructs were immersed in a mixture of 50% Stage 2 HLC differentiation medium and 50% Human Complete hSVF culture media (comprised of Medium 199 (Invitrogen), ECGS (derived in-house), HEPES, 1X L-glutamine (Invitrogen), and 10% heat-inactivated FBS (Thermo Fisher Scientific)). Constructs were implanted in the subcutaneous dorsa of isoflurane-anesthetized Rag1^-/-^ x LDLR^-/-^ double knockout mice (generously provided by Dr. Catherine Reardon-Alulis, University of Chicago). Animals were sacrificed at 2 weeks, at which point the implanted constructs were extricated and fixed in 4% PFA for 1h. Constructs were then permeabilized in 0.5% Triton X-100 (MP Biomedicals) for 20m and blocked in 5% Goat Serum (Invitrogen) for 1h. Afterwards, the explants were incubated overnight in Albumin (MP Biomedicals) and UEA-1 biotin (Vector Labs) at 1:200 in 5% Goat Serum at 4˚C. The following day, the constructs were washed in di-cation-free PBS and exposed to Goat-anti-Rabbit 488 (Thermo Fisher Scientific; for Albumin) and Streptavidin-Cy5 (Invitrogen; for UEA-1 biotin) at 1:1000 for 1h. Constructs were labeled with DAPI at 1:10000 for 10 minutes prior to confocal fluorescence microscopy imaging. All animal procedures were conducted in accordance with University of Louisville School of Medicine IACUC regulations.

**Polymerase Chain Reaction (PCR) and Gel Electrophoresis.** Differentiating FH-iPSC were lysed at the end of each stage using 350 μl of 0.1% β-mercaptoethanol in RLT Buffer (Qiagen). Lysates were purified using Qiashredder and RNeasy kits (Qiagen) according to the manufacturer’s instructions. RNA was quantified using a NanoDrop 1000 Spectrophotometer (generously provided by Ronald Gregg, University of Louisville). cDNA was generated from purified RNA via a SuperScript II Reverse Transcriptase kit (Invitrogen). 200 ng of stage-specific cDNA was added to 45 μl of PCR SuperMix (Invitrogen) and 1μl each of 10μM forward and reverse primers (Integrated DNA Technologies; primer sequences can be found in Supporting Information Table S1). PCR was performed for 25 cycles, with denaturation at 95˚C, annealing at 58˚C, and extension at 72˚C. Amplicons were assessed via 3% agarose gels (Bio-Rad). 10 μl of amplicons were combined with 2 μl of TrackIt™ Cyan/Yellow Loading Buffer (Invitrogen) and 2 μl of SYBR Green 1 nucleic acid stain (Lonza). Gels were run at 70 V for 2.5 hours and imaged via a Typhoon 9400 variable mode imager. Gel bands were plot profiled and normalized to GAPDH via densitometry analysis with Image J software (US National Institutes of Health).

**Immunocytochemistry.** For immunostaining, cells were fixed in 4% paraformaldehyde (PFA; Electron Microscopy Sciences) for 15 minutes at RT and washed with di-cation PBS. For intracellular iPSC staining, cells were permeabilized with 0.1% Triton X-100 (Sigma-Aldrich) and 1% Polyvinylpyrrolidone (Sigma-Aldrich), for iPSC analysis, in a PBS blocking solution containing 4% normal goat serum (Sigma-Aldrich). For extracellular staining, cells were blocked in di-cationic PBS containing 4% goat serum. Primary antibody solutions (1:200; Supporting Information Table S2) were applied for 1 hour at RT and washed cells with di-cation PBS. Secondary antibody solutions (1:500; Supporting Information Table S2) were applied for 1 hour at RT. Cells were treated with ProLong® Gold Antifade Reagent containing DAPI nuclear stain (Invitrogen). Images were taken using an Olympus IX81 fluorescence microscope using SlideBook Software (Intelligent Imaging Innovations, Inc., Denver, CO). To assess FH-iPSC differentiation into FH-HLC, cells were fixed and washed at the end of each stage as described above. After permeabilization with 0.5% Triton X-100 for 10 minutes, cells were blocked with 5% goat serum for 1 hour before being exposed to primary antibody overnight and secondary antibody the following day.

**Histology.** Explanted teratomas were fixed in paraffin (Leica Biosystems) and sectioned via microtome. Sections were de-paraffinized with xylene (Thermo Fisher Scientific) for 10 minutes and sequentially hydrated with decreasing proportions of ethanol in 2-minute increments. Sections were then immersed in water and exposed to Gill’s Hematoxylin (Leica Biosystems) for 10 minutes. Water was applied to the sections for 1 minute; sections were then dipped once each in acid ethanol (comprised of 3 ml 37% HCl (Thermo Fisher Scientific) and 300 ml 70% EtOH) and water before 15 immersions in ammonia water (0.9 ml 30% Ammonium Hydroxide; Sigma Aldrich) and exposure to running water for 20 minutes. Working Eosin (Leica Biosystems) was applied to sections for 5 minutes before sequential dehydration with increasing proportions of ethanol in 2-minute increments. After two more 2-minute immersions in xylene, sections were mounted with coverslips and imaged via phase microscopy.

**SUPPLEMENTAL REFERENCES**

1 Warren, L. *et al.* Highly efficient reprogramming to pluripotency and directed differentiation of human cells with synthetic modified mRNA. *Cell Stem Cell.* **7**, 618-630, doi:10.1016/j.stem.2010.08.012 (2010).

2 Takahashi, K. *et al.* Induction of pluripotent stem cells from adult human fibroblasts by defined factors. *Cell.* **131**, 861-872, doi:10.1016/j.cell.2007.11.019 (2007).

3 Boyer, L. A. *et al.* Core transcriptional regulatory circuitry in human embryonic stem cells. *Cell.* **122**, 947-956, doi:10.1016/j.cell.2005.08.020 (2005).

4 Krieger, M., Brown, M. S. & Goldstein, J. L. Isolation of Chinese Hamster Cell Mutants Defective in the Receptor-mediated Endocytosis of Low Density Lipoprotein. *J Mol Biol.* **150**, 167-184 (1981).

5 Hibbitt, O. C. *et al.* Long-term physiologically regulated expression of the low-density lipoprotein receptor in vivo using genomic DNA mini-gene constructs. *Mol Ther.* **18**, 317-326, doi:10.1038/mt.2009.249 (2010).

6 Ma, P. T. S. *et al.* Mevinolin, an inhibitor of cholesterol synthesis, induces mRNA for low density lipoprotein receptor in livers of hamsters and rabbits. *Proc Natl Acad Sci USA.* **83**, 8370-8374 (1986).

7 Ho, Y. K., Brown, M. S., Bilheimer, D. W. & Goldstein, J. L. Regulation of Low Density Lipoprotein Receptor Activity in Freshly Isolated Human Lymphocytes. *J Clin Invest.* **58**, 1465-1474 (1976).

8 Boyd, N. L., Robbins, K. R., Dhara, S. K., West, F. D. & Stice, S. L. Human Embryonic Stem Cell-Derived Mesoderm-Like Epithelium Transitions to Mesenchymal Progenitor Cells. *Tissue Eng Part A.* **15**, 1897-1907, doi:10.1089/ten.tea.2008.0351 (2009).

9 Mitalipova, M. M. *et al.* Preserving the genetic integrity of human embryonic stem cells. *Nat Biotechnol.* **23**, 19-20, doi:10.1038/nbt0105-19 (2005).

10 Hentze, H. *et al.* Teratoma formation by human embryonic stem cells: evaluation of essential parameters for future safety studies. *Stem Cell Res.* **2**, 198-210, doi:10.1016/j.scr.2009.02.002 (2009).

**SUPPLEMENTAL FIGURE & TABLE LEGENDS**

**Figure S1. Assessment of Source Fibroblast LDLR Deficiency.** Upon receipt, source FH fibroblasts (GM03155) were compared to wild-type IMR90 fetal lung fibroblasts to determine their ability to internalize fluorescent DiI-LDL after pre-treatment with Lovastatin, excess free sterols, or ethanol (vehicle control) in lipoprotein-deficient media. Internalization of fluorescently labeled LDL indicated a functional reduction in the capacity for receptor-mediated endocytosis.

**Figure S2. FH-iPSC Generation & Characterization. (A)** FH parent fibroblasts attained a compacted morphology characteristic of **(B)** iPSC, which were positive for **(C)** the pluripotent marker TRA-1-60 during live imaging. FH-iPSC were positive for **(D)** Oct4 and **(E)** Sox2, which **(F)** co-localized to the cell nucleus. FH-iPSC were also positive for the pluripotence markers **(G)** SSEA4, **(H)** TRA-1-60, and **(I)** TRA-1-81. Spontaneous *in vitro* differentiation yielded cells positive for **(J)** β-III TUBULIN (ectoderm), **(K)** VIMENTIN (endoderm), and **(L)** α-Smooth Muscle Actin (αSMA, mesoderm) (Scale bar = 10 μm). *In vivo* differentiation of implanted FH-iPSC yielded teratomas positive for **(M)** ectoderm, **(N)** mesoderm, and **(O)** endoderm.

**Figure S3. Karyotype Analysis. (A)** FH parent fibroblasts (GM01355) were tested to determine their karyotype. **(B)** After reprogramming, FH-iPSC karyotype was also determined. The karyotype of the starting population and reprogrammed daughter cells were both normal.

**Figure S4. Gene Transcript Quantification for Each Differentiation Stage.** Densitometry quantification of transcript expression was performed for each gene at the end of the differentiation stages. Transcripts were normalized to the GAPDH loading control. (Mean ± S.E.M)

**Figure S5. *pEHZ-LDLR-LDLR* functional confirmation.** Transfected CHO-a7-LDLR exhibited LDL internalization sensitive to Lovastatin, excess sterols, or ethanol (vehicle control). This behavior paralleled that of CHO-WT under the same media conditions, and both were in stark contrast to the lack of LDL-C uptake seen in *Ldlr*-dysfunctional CHO-a7.

**TABLE S1. PCR Primers.** A listing of the primers used for RT-PCR.

**TABLE S2. Antibodies and Flurophores.** Primary antibodies were incubated at 1:200, Fluorophores at 1:500, and secondary antibodies at 1:1000.

**SUPPLEMENTAL FIGURE S1**


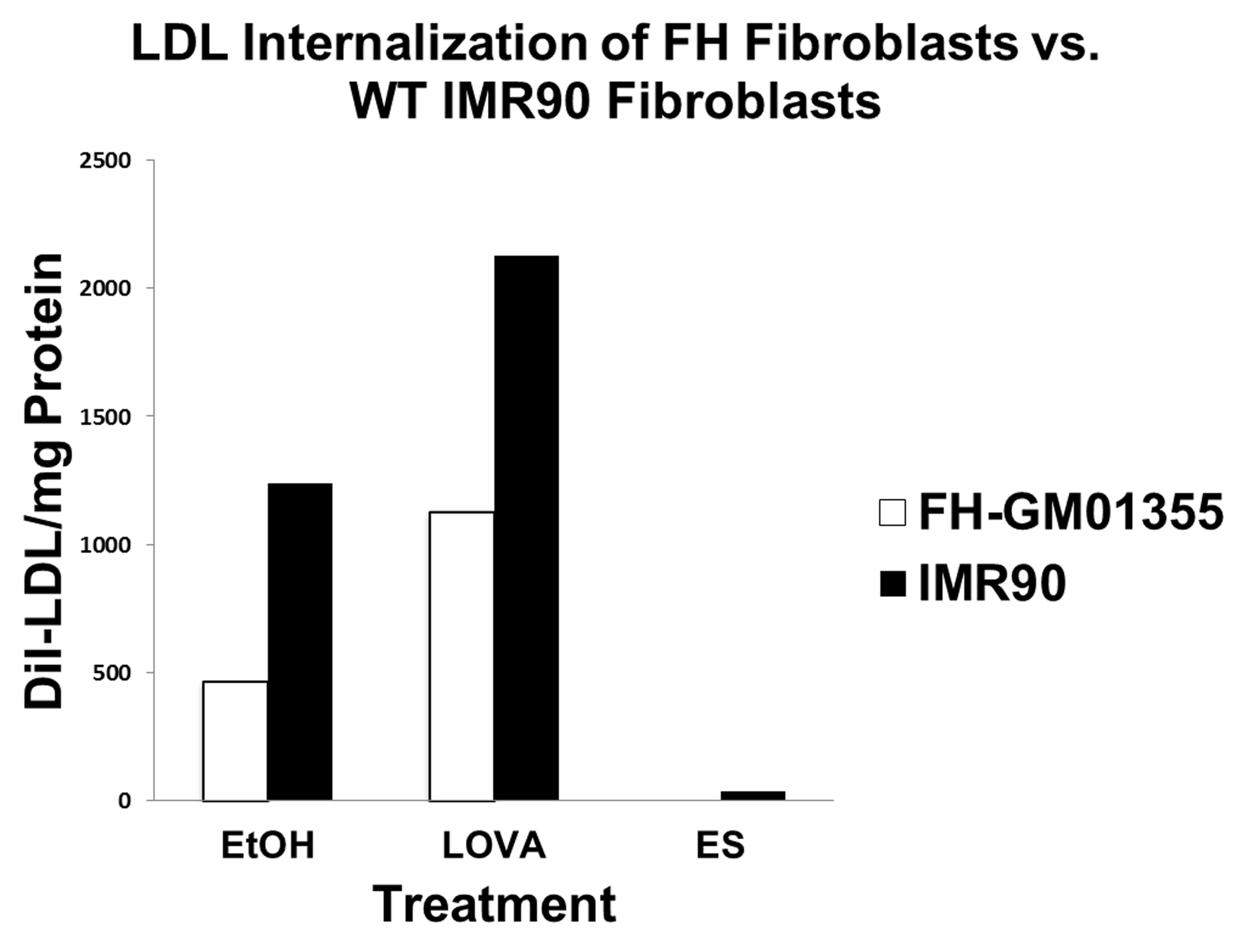


**SUPPLEMENTAL FIGURE S2**

**
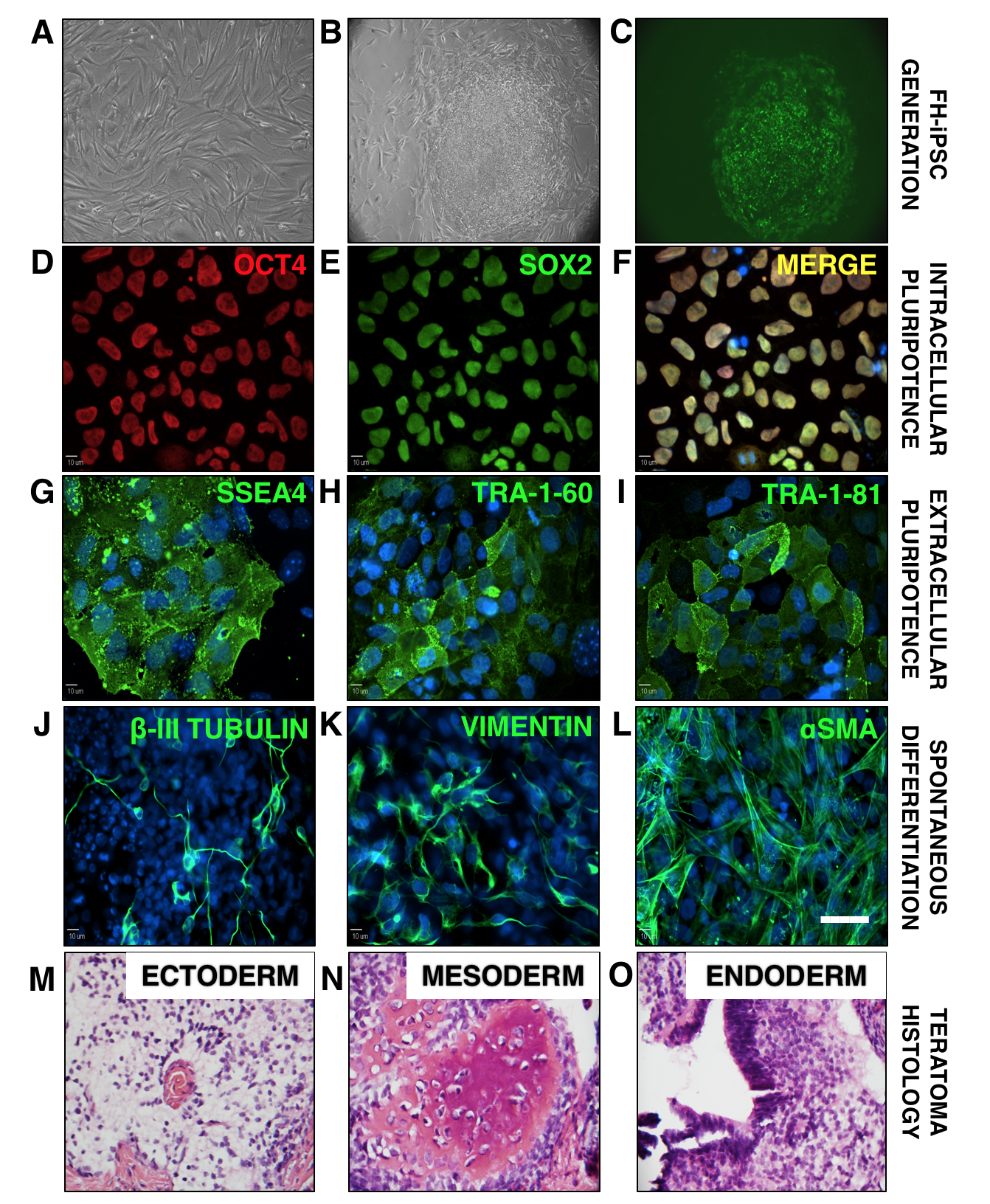
**

**SUPPLEMENTAL FIGURE S3**

**A** - FH Source Fibroblasts (GM01355)

**
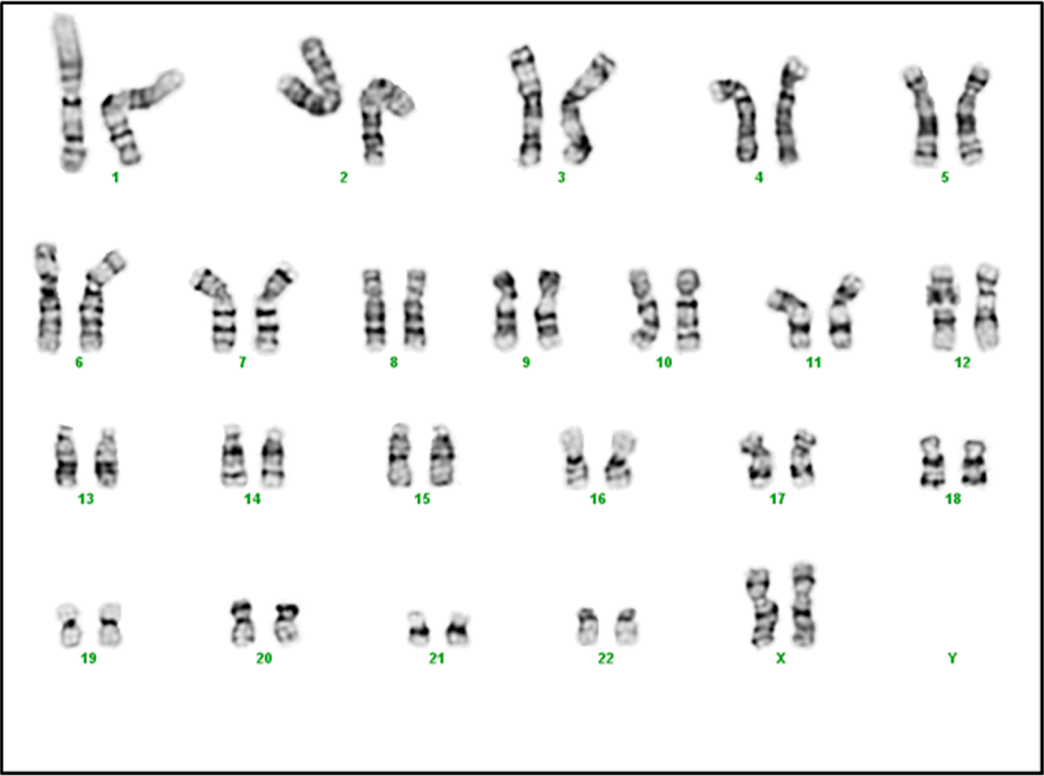
**

**B** – FH-iPSC

**
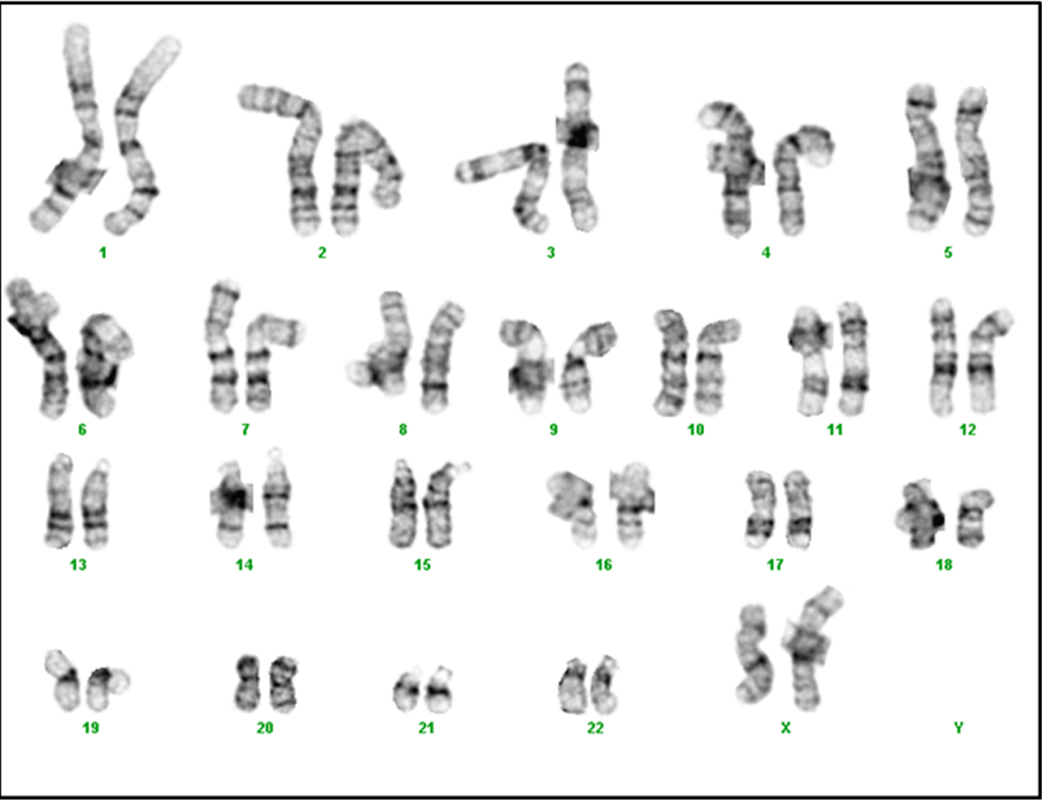
**

**SUPPLEMENTAL FIGURE S4**

**
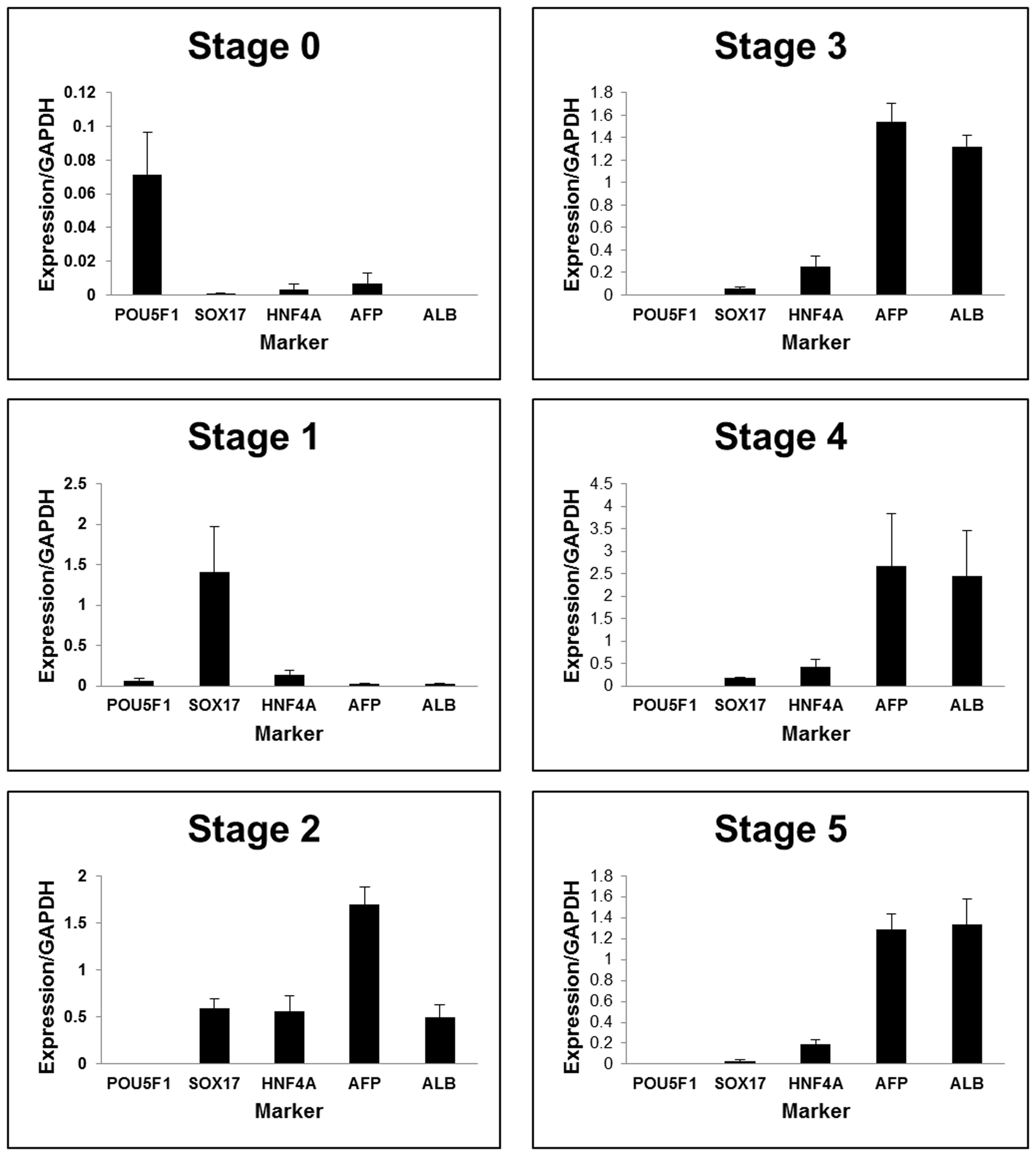
**

**SUPPLEMENTAL FIGURE S5**

**
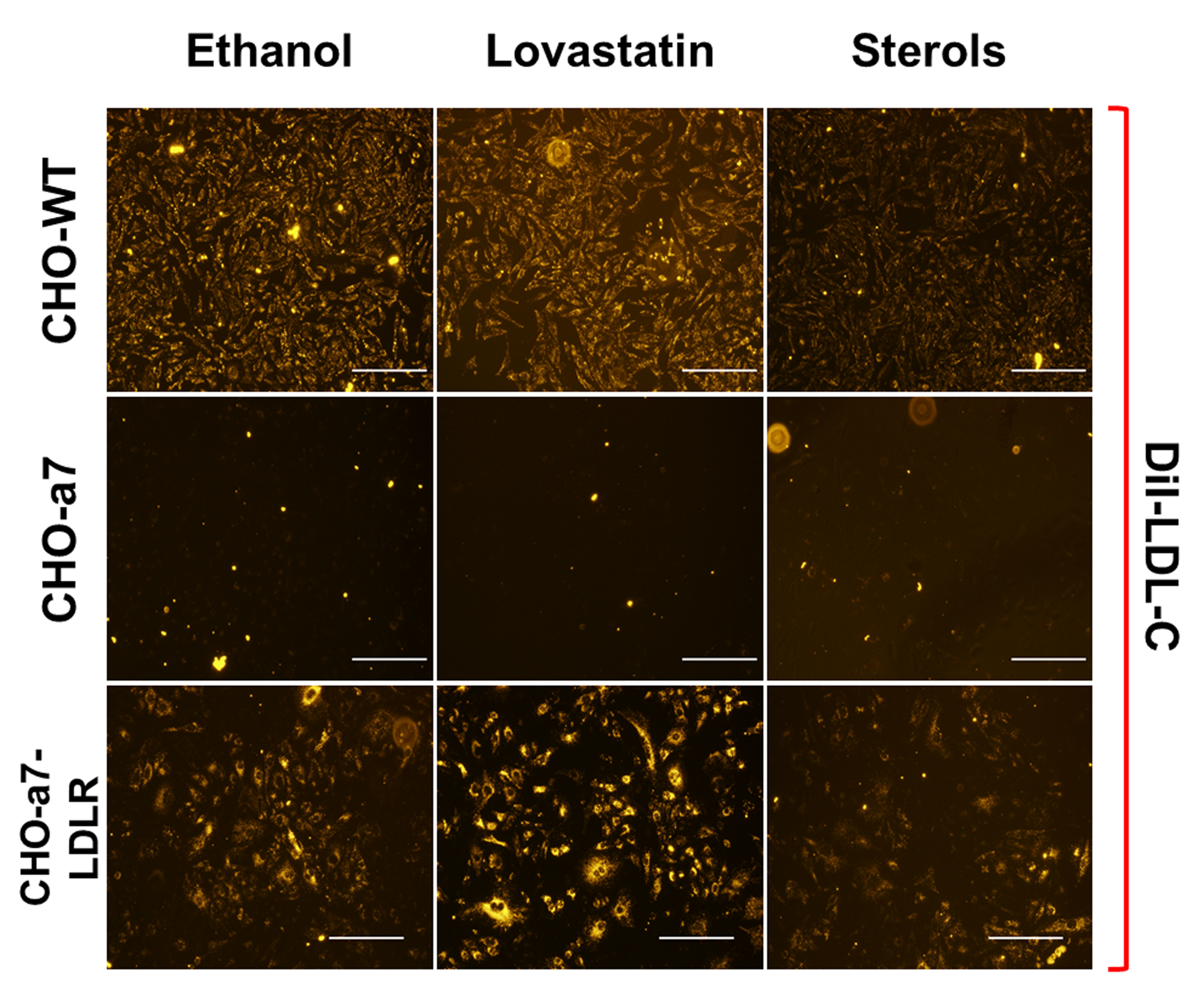
**

**SUPPLEMENTAL TABLE S1**

**List of Primers Used in PCR**

| **Gene Name** | **Primer Sequence**  **(5’ 🡪 3’)** | **Product Size (bp)** | **Primer-BLAST Annealing Temperature** |
| --- | --- | --- | --- |
| **POU5F1** | Forward: TGATCCTCGGACCTGGCTAA  Reverse:  AACCACACTCGGACCACATC | 744 | Forward: 60.03˚C  Reverse: 59.97˚C |
| **SOX17** | Forward:  CCGCGGTATATTACTGCAACTA  Reverse:  CACCCAGGACAACATTTCTTTG | 371 | Forward:  58.1˚C  Reverse: 58.02˚C |
| **HNF4a** | Forward:  CCACGGGCAAACACTACGG  Reverse:  GGCAGGCTGCTGTCCTCAT | 250 | Forward: 61.32˚C  Reverse: 62.34˚C |
| **AFP** | Forward:  GCTGACCTGGCTACCATATTT  Reverse:  GGGATGCCTTCTTGCTATCTC | 384 | Forward:  57.8˚C  Reverse:  58.2˚C |
| **ALB** | Forward:  GGTGTTGATTGCCTTTGCTC  Reverse:  CCCTTCATCCCGAAGTTCAT | 502 | Forward: 57.93˚C  Reverse: 57.28˚C |
| **NANOG** | Forward:  TCTGCTGGACTGAGCTGGTT  Reverse:  GTGCACCAGGTCTGAGTGTT | 866 | Forward:  61.12˚C  Reverse:  60.18˚C |
| **β-ACTIN** | Forward:  CTGTGGCATCCACGAAACTA  Reverse:  AGTACTTGCGCTCAGGAGGA | 200 | Forward:  54.94˚C  Reverse:  58.03˚C |
| **GAPDH** | Forward:  AATCCCATCACCATCTTCC  Reverse:  CATCACGCCACAGTTTCC | 382 | Forward: 54.14˚C  Reverse: 56.43˚C |

**SUPPLEMENTAL TABLE S2**

**List of Antibodies and Fluorophores**

**Verification of pluripotence**

| **PRIMARY ANTIBODY (1:200)** | **MANUFACTURER & LOCATION** |
| --- | --- |
| OCT4 | R&D Systems, Minneapolis, MN |
| SOX2 | R&D Systems |
| SSEA4 | Developmental Studies Hybridoma Bank, Iowa City, IA |
| TRA-1-60 | EMD Millipore, Billerica, MA |
| StainAlive^TM^ TRA-1-60 (DyLight^TM^ 488) | Stemgent, Cambridge, MA |
| TRA-1-81 | EMD Millipore |
| βIII-TUBULIN | Neuromics, Edina, MN |
| α – SMOOTH MUSCLE ACTIN | Santa Cruz Biotechnology, Dallas, TX |
| VIMENTIN | BD Bioscience, San Jose, CA |

| **FLUOROPHORE (1:500)** | **MANUFACTURER & LOCATION** |
| --- | --- |
| Alexa Fluor® 405 | Invitrogen, Grand Island, NY |
| Alexa Fluor® 594 | Invitrogen |

**End-of-stage visual assessments**

| **PRIMARY ANTIBODY (1:200)** | **MANUFACTURER & LOCATION** |
| --- | --- |
| OCT4 | Santa Cruz Biotechnology |
| SOX17 | R&D Systems |
| GATA4 | Santa Cruz Biotechnology |
| HNF3β | Santa Cruz Biotechnology |
| AFP (ZSA06) | Invitrogen |
| Albumin | MP Biomedicals, Santa Ana, CA |

| **SECONDARY ANTIBODY (1:1000)** | **MANUFACTURER & LOCATION** |
| --- | --- |
| Goat anti-Mouse 488 | Thermo Scientific, Waltham, MA |
| Goat anti-Rabbit 488 | Thermo Scientific |
| Rabbit anti-Goat 594 | Invitrogen |
